# High overexpression of fatty acid synthase is associated with poor survival in Chinese patients with gastric carcinoma

**DOI:** 10.3892/etm.2012.727

**Published:** 2012-09-27

**Authors:** WENMIN HOU, MAOGUI FEI, XIA QIN, XUEHUA ZHU, JOEL GRESHOCK, PING LIU, YUANFENG ZHOU, HUI WANG, BANG-CE YE, CRYSTAL YING QIN

**Affiliations:** 1 Laboratory of Biosystems and Microanalysis, State Key Laboratory of Bioreactor Engineering, East China University of Science and Technology;; 2 Key Laboratory of Nutrition and Metabolism, Institute for Nutritional Sciences, Shanghai Institutes for Biological Sciences, Chinese Academy of Sciences, Graduate School of the Chinese Academy of Sciences;; 3 Department of Oncology, GlaxoSmithKline Research and Development Center;; 4 Shanghai Key Laboratory of Signaling and Disease Research, School of Life Science and Technology, Tongji University, Shanghai, P.R. China;; 5 Cancer Research, GlaxoSmithKline, Collegeville, PA, USA

**Keywords:** fatty acid synthase, gastric cancer, tissue microarray, prognostic marker

## Abstract

Fatty acid synthase (FAS) is the key enzyme regulating *de novo* biosynthesis of fatty acids. FAS overexpression has been found in many types of tumors and is associated with poor survival. However, the expression of FAS and its relationship with prognosis in Chinese patients with gastric carcinoma are still unknown. Therefore, in this study, we examined the expression of FAS using tissue microarrays and determined its correlation with clinicopathological characteristics and prognosis of gastric carcinoma in Chinese patients. FAS overexpression was graded as S (T/A) <1, ≥1 to <2, ≥2 to <3 or ≥3 in 35 (38.9%), 20 (22.2%), 9 (10%) and 26 (28.9%) patients, respectively. High FAS overexpression [S (T/A) ≥3] was significantly correlated with poor prognosis (log-rank test, P= 0.0078) and with decreased 3-year survival rate (χ^2^ test, P=0.0023). FAS overexpression was not significantly associated with other clinicopathological characteristics. In conclusion, our results suggest that FAS expression might be a potential prognostic marker for gastric carcinoma in Chinese patients.

## Introduction

Fatty acids (FAs) are biological molecules with important physiological roles in energy storage, membrane formation and protein acylation (1). Animals acquire FAs from the diet and via *de novo* biosynthesis (2). In the latter, FAs are predominantly generated by a 250- to 270-kDa multifunctional and homodimeric enzyme, fatty acid synthase (FAS). Long-chain FAs, the main product of FAS, are derived from acetyl-CoA, malonyl-CoA and NADPH (3). FAs are essential constituents of biological membranes and are important substrates in energy metabolism. Although the mechanisms responsible for FAS overexpression in tumors are not fully understood, the PTEN/PI3K/AKT and RAS/RAF/MAPK/ERK1/2 pathways are known to regulate FAS expression (3,4), and these pathways are often hyperactive in tumors. Notably, in the LNCaP prostate cancer cell line, pharmacological inhibition of PI3K or reintroduction of wild-type PTEN was found to reduce FAS expression (4).

Most tissues, except for the liver, adipose tissue, cycling endometrium (5), fetal lungs (6), lactating breast (7,8) and embryos (3,9) utilize dietary FAs to build new structural lipids. Therefore, FAS is expressed at low levels in most normal tissues. By contrast, in cancer tissues, the FA supply is highly dependent on *de novo* biosynthesis via FAS. Indeed, several studies have shown that FAS is overexpressed in many cancers, including breast (10,11), prostate (12,13), ovarian (14) and colorectal carcinomas (15,16). Furthermore, high FAS expression is associated with advanced clinical stage, poor differentiation and poor prognosis of breast (10), prostate (17) and ovarian carcinomas (15). Downregulation of FAS by RNAi was found to inhibit growth and apoptosis in LnCaP cells but not in normal fibroblasts (18). Furthermore, pharmacological or RNAi-mediated downregulation of FAS significantly sensitized the responsiveness of breast cancer cell lines (SK-Br3, MCF-7 and MDA-MB-231) to paclitaxel or vinorelbine (19,20). These results indicate that FAS is an important prognostic factor in certain types of cancers and may represent a potential therapeutic target for cancer chemotherapy.

However, FAS expression in gastric carcinoma, one of the most prevalent malignant tumors worldwide, particularly in China, has not been established. To date, few clinical studies have determined FAS expression in gastric carcinoma or compared its expression with that in non-neoplastic adjacent tissue (21,22). Since FAS expression varies at different ages and clinical circumstances, determining FAS expression in tumor tissue alone is insufficient to clarify the prognostic relevance of FAS expression in cancer. Therefore, to provide insight into the clinical relevance of FAS, we examined FAS expression in gastric carcinoma and paired adjacent normal tissue samples collected from 90 Chinese patients. We analyzed the associations between FAS expression and clinicopathological characteristics, such as age, gender, histological grade, American Joint Committee on Cancer (AJCC) tumor stage, metastasis and tumor size, as well as molecular markers, such as the loss of PTEN and pERK1/2 expression. Finally, we determined the effects of FAS expression on prognosis.

## Materials and methods

### Patients and tissue samples

Ninety patients with gastric carcinoma who underwent surgery between 2007 and 2008 were enrolled in this study. None of the patients had received any treatment before surgery. We obtained complete clinicopathological information for all patients, including age, gender, tumor size, histological grade, AJCC tumor stage, depth of invasion, lymph node metastasis and distant metastasis. All of the patients included in this study had adenocarcinoma. The median age of the patients at the time of diagnosis was 65.5 years (range 34–83 years). The histological grade of the tumor was evaluated based on the degree of tumor differentiation, tumor necrosis and mitotic count, according to the criteria of Enzinger and Weiss (23). Follow-up time was calculated as the time from initial surgery to the death of the patient due to the primary tumor or the date of last contact. Tumor tissue and paired adjacent normal tissue samples were obtained at surgery. All tissues were dissected in the operating room, immediately frozen, and stored at −80°C. Informed consent for use of tissue samples in future molecular studies was obtained from each patient. This study was approved by the Ethics Committee of the Third Xiangya Hospital, Central South University (Hunan, China). Clinical and treatment information was extracted by chart review carried out by the surgeon with approval from our institutional review board.

### Tissue microarray (TMA) preparation

Core needle biopsies (1.5 mm diameter) were extracted from paraffin-embedded tissue samples, and mounted into a recipient paraffin block using a dedicated tissue array instrument (Beecher Instruments, Sun Prairie, WI, USA). Then, 4-*μ*m-thick sections of the TMA were cut, transferred to glass slides and stained with hematoxylin and eosin.

### Immunohistochemistry

TMA sections (1.5-mm diameter; 4-*μ*m thick) from archival, formalin-fixed, paraffin-embedded tissue specimens were mounted on poly-L-lysine (Muto Chemicals, Tokyo, Japan)-coated slides. The TMA sections were deparaffinized in xylene for 15 min, rehydrated in an ethanol gradient and heated at 95°C for 5 min in 10 mM sodium citrate buffer (pH 6.0) in a microwave oven for antigen retrieval. Endogenous peroxidase was inactivated by incubating the sections in 3% H_2_O_2_ for 15 min at room temperature. The sections were blocked in 3% normal donkey serum and incubated at 4°C overnight with monoclonal anti-FAS antibody (dilution, 1:50; no. 3180S, Cell Signaling Technology, Danvers, MA, USA), anti-pERK1/2 antibody (dilution, 1:1000; no. 4370, Cell Signaling Technology) and anti-PTEN antibody (dilution, 1:50; no. 9559C, Cell Signaling Technology). Finally, the sections were stained with horseradish peroxidase-conjugated donkey anti-rabbit IgG (H+L) secondary antibody (711-035-152, Jackson ImmunoResearch Europe, Newmarket, UK). Signal detection was carried out using a Dako signaling amplification system (K346811; Dako, Glostrup, Denmark). The TMA sections were counterstained with hematoxylin, dehydrated, and mounted.

### TMA score

Immunohistochemistry was scored based on staining intensity and the percentage of positive cells. The staining intensity was scored as follows: 0, negative; 1, weak; 2, moderate; and 3, high intensity. The immunoreactive score was calculated as staining intensity score x percentage of FAS-positive cells. We also calculated S (T/A), immunore-active score of tumor tissue/immunoreactive score of paired adjacent normal tissue, as an index for the difference in expression between tumor and normal tissue. FAS immunostaining was analyzed under a microscope (Nikon Eclipse E600) and estimated independently by two pathologists.

### Statistical analysis

Statistical analyses were performed using GraphPad Prism 5 (GraphPad Software Inc., San Diego, CA, USA) for Windows. The Student’s t-test was used to compare FAS expression between cancer tissue and adjacent normal tissues. Contingency table analysis and χ^2^ tests were used to investigate the relationship between FAS expression and clinical variables. For outcomes with a small number of cases, Fisher’s exact test was used. Survival was estimated using the Kaplan-Meier method, and differences in survival curves were determined using the log-rank test. Values of P<0.05 were regarded as statistically significant.

## Results

### Clinicopathological characteristics

Table I summarizes the clinicopathological characteristics data of the patients with previously untreated gastric carcinoma. The study cohort included 67 males and 23 females, ranging in age from 34 to 83 years (median age, 65 years). The histological type of all patients was adenocarcinoma. Tumor size ranged from 0 to 20 cm with a mean size of 6.17 cm and a median size of 5.75 cm. The tumors were classified as grade I in 1 patient, grade II in 23 patients and grade III in 66 patients. The median and mean duration of follow-up was 35 and 31.75 months, respectively, ranging from 1 to 51 months. Most of the patients (77.8%, 70/90) developed distant or regional lymph node metastasis.

### FAS expression

**Figure 1:**
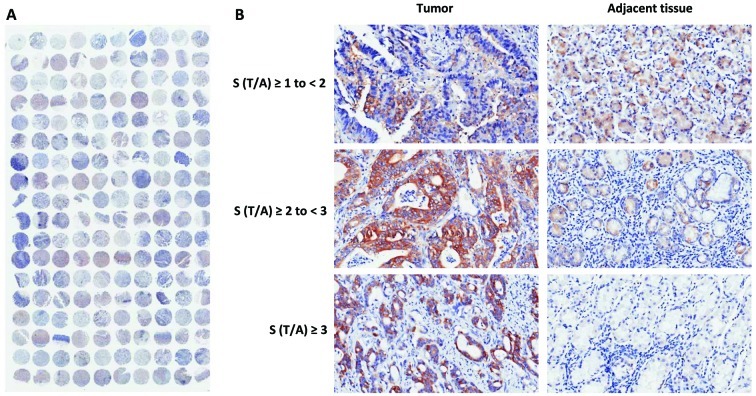
Immunohistochemical expression of FAS in gastric carcinomas. (A) Representative tissue microarray. The odd and even lines represent tumor and adjacent normal tissue samples, respectively. (B) Representative cases of immunohistochemical expression of FAS for S (T/A) ≥1 to <2, S (T/A) ≥2 to <3 and S (T/A) ≥3. S (T/A) = immunohistochemistry score (tumor)/immunohistochemistry score (adjacent tissue). Immunohistochemistry score = staining intensity x percent of FAS-positive cells. Staining intensity was scored as 0, negative; 1, weak; 2, moderate; and 3, high.

**Figure 2:**
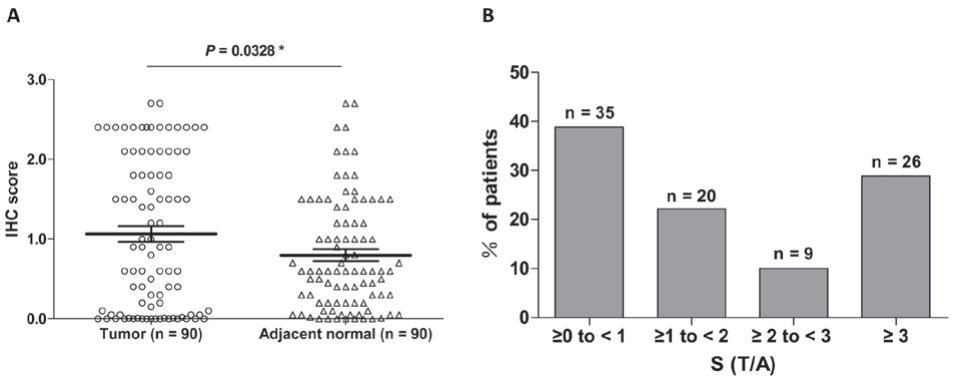
(A) Scatter plot showing the distribution of the immunohistochemical (IHC) score for FAS expression in gastric tumor tissues and adjacent normal tissues. The mean IHC scores for FAS expression in tumors and adjacent tissues are 1.065±0.099 and 0.7981±0.074, respectively. The P-value was determined by an unpaired test. (B) Distribution of FAS expression defined as S (T/A). Overall, 38.9% (35/90), 22.2% (20/90), 10% (9/90) and 28.9% (26/90) of the carcinoma tissue specimens were classified as S (T/A) ≥0 to <1, S (T/A) ≥1 to <2, S (T/A) ≥2 to <3 and S (T/A) ≥3, respectively.

We determined FAS expression in all 90 tumor tissue and paired adjacent normal tissue samples by TMA and immunohistochemistry. A representative TMA stained for FAS is shown in Fig. 1A. No signal was detected in the nuclei or on the cell membrane, indicating that FAS was mainly localized to the cytoplasm. The mean immunohistochemical score of FAS expression was significantly higher in tumor tissue than in adjacent normal tissue (1.065±0.099 vs. 0.798±0.074, P<0.05, Fig. 2A). These data indicate that FAS was overexpressed in the cytoplasm in tumor tissue compared to the expression in the adjacent normal tissue. To investigate the difference in expression between tumor tissues and normal tissues, we calculated S (T/A) as described in Materials and methods. Representative tissue sections corresponding to S (T/A) ≥1 to <2 (low), S (T/A) ≥2 to <3 (medium) and S (T/A) ≥3 (high expression) are shown in Fig. 1B. Overall, 38.9% (35/90), 22.2% (20/90), 10% (9/90) and 28.9% (26/90) of the carcinoma tissue specimens were classified as S (T/A) ≥0 to <1, S (T/A) ≥1 to <2, S (T/A) ≥2 to <3 and S (T/A) ≥3, respectively (Fig. 2B).

### Relationship between FAS overexpression and clinicopathological parameters

To investigate the association between FAS overexpression and clinicopathological parameters, three grades of FAS overexpression (S (T/A) ≥1, ≥2 and ≥3) were established. FAS overexpression was not significantly associated with any of the clinicopathological variables recorded, including age, gender, grade, tumor size and lymph node metastasis (Table II). As several reports have shown that FAS expression is modulated by the PTEN/PI3K/AKT and RAS/RAF/MEK/ERK pathways, we determined the expression of several components in these two pathways (4,24). Surprisingly, FAS overexpression was not significantly correlated with total, cytoplasmic or nuclear loss of PTEN (Table II) or pERK expression (data not shown) in gastric carcinomas.

### Survival analysis

**Figure 3:**
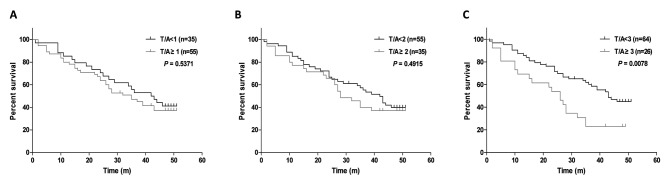
The highest level of FAS overexpression is associated with poor survival of patients with gastric carcinoma. (A–C) show survival curves for patients with carcinomas classified as S (T/A) ≥1, ≥2 and ≥3, respectively. FAS overexpression corresponding to S (T/A) ≥1 or ≥2 was not associated with survival. Patients with S (T/A) ≥3 had significantly worse overall survival compared with all other patients (log-rank test, P=0.0078).

Finally, we conducted survival analysis to determine whether FAS overexpression in tumors was associated with survival by plotting Kaplan-Meier survival curves and calculating the 3-year survival rate for all three grades of FAS overexpression. For cases with S (T/A) ≥1 or ≥2, FAS overexpression was not associated with overall survival or 3-year survival rate (Table III; Fig. 3A and B). However, among cases with S (T/A) ≥3, FAS overexpression was significantly associated with poor survival (Fig. 3C, log-rank test, P=0.0078) and with a decreased 3-year survival rate (Table III; χ^2^ test, P=0.0023).

## Discussion

FAS protein, also known as oncogenic antigen 519 (OA-519), is overexpressed and hyperactivated in the majority of human malignancies. It plays a central role in the maintenance of the malignant phenotype by enhancing cancer cell survival and proliferation (3). Overexpression of FAS has been reported in human carcinomas including prostate, ovary, breast, colon, endometrium, thyroid gland, squamous cell carcinoma of the lung, and gastric carcinomas. In this study, FAS overexpression was detected in 22.2% (20/90), 10% (9/90) and 28.9% (26/90) of the carcinoma tissue specimens graded as S (T/A) ≥1 to <2, S (T/A) ≥2 to <3 and S (T/A) ≥3, respectively.

Many studies have revealed that FAS overexpression and hyperactivity is regulated by the MAPK/ERK1/2 and PTEN/PI3K/AKT signaling pathways. Therefore, we examined PTEN and pERK expression levels in the same TMA samples. However, FAS overexpression was not correlated with pERK expression or the loss of PTEN in these gastric carcinoma specimens. These findings suggest that there are signaling pathways independent of the PTEN/PI3K/AKT and RAS/RAF/MEK/ERK pathways, such as the ubiquitin-protease pathway, that regulate FAS expression (25).

Although FAS overexpression at any of the three levels defined in this study was not associated with any of the clinicopathological parameters assessed (age, gender, AJCC stage and histological grade), high FAS overexpression [S (T/A) ≥3] was significantly associated with poor survival and with a reduced 3-year survival rate. Although several studies have reported that FAS is overexpressed in gastric cancer (21,22), to our knowledge, our study is the first to show that high FAS overexpression could be a prognostic marker in gastric carcinoma.

In addition to its potential utility as a prognostic marker for cancer patients, FAS shows some promise as a chemotherapeutic target (3,26–28). For example, several studies have shown that cerulenin, a specific noncompetitive inhibitor of the β-ketoacyl synthase activity of FAS, is selectively cytotoxic to breast and ovarian cancer cells exhibiting enhanced fatty acid synthesis, but not to normal cells with constitutively low FAS expression (29,30). Based on these and other results, more research into the clinical relevance of FAS overexpression is necessary, particularly because FAS may represent an excellent target for treating gastric carcinoma, and other tumors.

## Figures and Tables

**Table I:** TMA clinical information.

Characteristics	
Gender, n (%)	
Male	67 (74.4)
Female	23 (25.6)
Age (years)	
Median (range)	65 (34–83)
Histological type, n (%)	
Adenocacinoma	90 (100.0)
Others	0 (0.0)
Presentation, n (%)	
Initial	90 (100.0)
Recurrent	0 (0.0)
Size (cm)	
Median (range)	5.75 (0–20)
Grade, n (%)	
I	1 (1.1)
II	23 (25.6)
III	66 (73.3)
Metastasis, n (%)	
Negative	20 (22.2)
Positive	70 (77.8)

**Table II:** FAS expression and clinicopathological factors of the gastric carcinoma patients.

	S (T/A) <1	S (T/A) ≥1	^a^P-value	S (T/A) ≥2	^b^P-value	S (T/A) ≥3	^c^P-value
Age (years), n (%)							
Median age	65	65		69		65	
Range	45–83	34–83		41–81		41–81	
<60	14 (48.3)	15 (51.7)	0.2078	7 (24.1)	0.0645	6 (20.7)	0.6086
≥60	21 (34.4)	40 (65.6)		28 (45.9)		20 (32.8)	
Gender, n (%)							
Female	6 (26.1)	17 (73.9)	0.1444	12 (52.2)	0.1298	9 (39.1)	0.286
Male	29 (43.3)	38 (56.7)		23 (34.3)		17 (25.4)	
Histological grade, n (%)							
I	0 (0.0)	1 (100.0)	0.4328	0 (0.0)	0.4524	0 (0.0)	0.7564
II	7 (30.4)	16 (69.6)		11 (47.8)		6 (26.1)	
III	28 (42.4)	38 (57.6)		24 (36.4)		20 (30.3)	
AJCC tumor stage, n (%)							
I	1 (16.7)	5 (83.3)	0.5244	2 (33.3)	0.4868	1 (16.7)	0.2082
II	12 (40.0)	18 (60.0)		11 (36.7)		6 (20.0)	
III	20 (39.2)	31 (60.8)		22 (43.1)		19 (37.3)	
IV	2 (66.7)	1 (33.3)		0 (0.0)		0 (0.0)	
Metastasis, n (%)							
Negative	5 (25)	15 (75.0)	0.1485	8 (40.0)	0.908	5 (25.0)	0.6635
Positive	30 (42.9)	40 (57.1)		27 (38.6)		21 (30.0)	
Tumor size (cm), n (%)							
<5	14 (45.2)	17 (54.8)	0.3763	11 (35.5)	0.631	7 (22.6)	0.3385
≥5	21 (35.6)	38 (64.4)		24 (40.7)		19 (32.2)	
PTEN (total), n (%)							
Negative	12 (36.4)	21 (63.6)	0.8234	13 (39.4)	1.0	10 (30.3)	0.8145
Positive	23 (40.4)	34 (59.6)		22 (38.6)		16 (28.1)	
PTEN (cytopasmic), n (%)							
Negative	24 (39.3)	37 (60.7)	1.0	23 (37.7)	0.8184	16 (26.2)	0.4612
Positive	11 (37.9)	18 (62.1)		12 (41.4)		10 (36.7)	
PTEN (nuclear), n (%)							
Negative	14 (35.0)	26 (65.0)	0.5224	16 (40.0)	1.0	12 (30.0)	1.0
Positive	21 (42.0)	29 (58.0)		19 (38.0)		14 (28.0)	

P-value is determined by χ^2^ exact test,

a S (T/A) <1 vs. S (T/A) ≥1,

b S (T/A) <2 vs. S (T/A) ≥2,

c S (T/A) <3 vs. S (T/A) ≥3.

**Table III:** FAS expression and 3-year overall survival of the gastric carcinoma patients.

Overall survival	S (T/A) <1	S (T/A) ≥1	S (T/A) <2	S (T/A) ≥2	S (T/A) <3	S (T/A) ≥3
≥3 years	18	26	29	15	38	6
<3 years	17	29	26	20	26	20
P-value	0.8292		0.3939		0.0023	

P-value is determined by χ^2^ exact test.
